# Adequate Dietary Intake and Vitamin D Supplementation: A Study of Their Relative Importance in Determining Serum Vitamin D and Ferritin Concentrations during Pregnancy

**DOI:** 10.3390/nu14153083

**Published:** 2022-07-27

**Authors:** Rosa S. Wong, Keith T. S. Tung, Yannis W. K. Chan, Bianca N. K. Chan, Wing-Cheong Leung, Jason C. Yam, Patrick Ip

**Affiliations:** 1 Department of Paediatrics and Adolescent Medicine, The University of Hong Kong, Hong Kong, China; rosawg@connect.hku.hk (R.S.W.); keith-tung@connect.hku.hk (K.T.S.T.); yannisschan23@gmail.com (Y.W.K.C.); bbianca@hku.hk (B.N.K.C.); 2 Department of Social Work and Social Administration, The University of Hong Kong, Hong Kong, China; 3 Department of Pharmacology and Pharmacy, The University of Hong Kong, Hong Kong, China; 4 Department of Obstetrics and Gynaecology, Kwong Wah Hospital, Hong Kong, China; leungwc@ha.org.hk; 5 Department of Ophthalmology and Visual Sciences, The Chinese University of Hong Kong, Hong Kong, China; yamcheuksing@cuhk.edu.hk

**Keywords:** vitamin D, pregnancy, supplementation, dietary patterns, 25-hydroxyvitamin D

## Abstract

Vitamin D is essential for human health. However, it is not clear if vitamin D supplementation is necessary for all pregnant women. This study examines the relative importance of dietary patterns and vitamin D supplementation frequency in determining serum 25-hydroxyvitamin D (25(OH)D) and ferritin concentrations among pregnant women in Hong Kong, China. A total of 572 healthy women were recruited from antenatal clinics at 25–35 weeks pregnant. Participants completed an electronic version of the food frequency questionnaire and a web questionnaire on supplement use. Their blood samples were tested for serum 25(OH)D and ferritin. The associations of dietary patterns and vitamin D supplementation frequency with serum 25(OH)D and ferritin concentrations were analyzed using moderated hierarchical regression. Two dietary patterns were identified. The adequate dietary intake was characterized by the high probability of meeting recommended daily food group servings, whereas the inadequate dietary intake was characterized by inadequate consumption of vegetables, fruits, meat, fish, and eggs, or alternatives. The association between adequate dietary intake and serum ferritin concentrations was independent of vitamin D supplementation frequency (β = 0.05, *p* = 0.035), but dietary patterns interacted with vitamin D supplementation frequency to determine serum 25(OH)D concentrations (β = −13.22, *p* = 0.014). The current study presents evidence on the relative importance of dietary patterns and vitamin D supplementation in maintaining sufficient vitamin D and iron in pregnancy. Antenatal nutrition counselling services should be provided to pregnant women who show signs of inadequate dietary intake.

## 1. Introduction

Vitamin D is a steroid hormone that helps to maintain calcium homeostasis and facilitates bone mineralization in the body. Vitamin D deficiency during pregnancy can cause bone loss, preeclampsia, preterm delivery, and low birth weight [1]. Iron-deficiency anemia is another common nutritional-deficiency disease during pregnancy. Under normal circumstances, serum ferritin concentrations can be indicative of the total amount of iron stored in the body. While there are many iron-rich foods such as red meat, livers, nuts, and green, leafy vegetables, the food sources of vitamin D are relatively limited [2]. In humans, vitamin D is primarily produced from the skin by way of exposure to ultraviolet light. However, due to the increased nutritional needs for body metabolism and fetal growth, pregnant women are advised to take daily vitamin D supplements [3]. A previous study showed that the most effective and safest dose for achieving optimal serum vitamin D levels among pregnant women in all races is 4000 IU per day [4]. It is however not clear if vitamin D supplementation is still required for healthy women who have adequate dietary intake to obtain sufficient vitamin D during pregnancy.

Previous research has shown no meaningful differences in iron status improvement among women receiving different doses of vitamin D supplementation during gestation (4200 IU, 16,800 IU, and 28,000 IU per week) [5]. While some evidence shows that vitamin D supplementation cannot improve serum ferritin concentrations in pregnant women [5,6,7], low vitamin D status was found to be associated with an increased risk of prenatal iron deficiency [8]. Notably, although previous research has examined the effect of vitamin D supplementation on both vitamin D and ferritin concentrations, it remains unclear whether vitamin D supplementation could have simultaneous effects on both vitamin D and iron levels. Furthermore, while natural food are important sources of iron, little work has been undertaken to examine the effect of diet and its potential interaction with vitamin D supplementation on vitamin D status, particularly during pregnancy.

Hong Kong provides a good setting for research to examine the relative effects of diet and supplementation on vitamin D concentrations because of its small changes in sunlight intensity between months and sunlight exposure duration between individuals. Thus, when the research is conducted in Hong Kong, the effects of sunlight exposure on vitamin D status can be minimized. Furthermore, given the nutritional benefits of natural foods and the amount of nutrients needed during pregnancy, the dietary intake recommended by Hong Kong’s local health agencies for pregnant women who are in the second and third trimesters (14th to 40th week), have a normal body mass index, and weigh between 45 and 60 kg before pregnancy is characterized by five food groups including grains (3.5–5 servings per day); vegetables (4–5 servings per day); fruits (2–3 servings per days); meat, fish, eggs, or alternatives (5–7 servings per day); and dairy or alternatives (2 servings per days) [9]. Local researchers can use these recommended daily servings as the reference threshold levels to classify pregnant women who have adequate dietary intake and those who do not in the population. The objective of this study is thus to examine the relative importance of dietary patterns (adequate/inadequate dietary intake) and vitamin D supplementation frequency (at least/less than 1 time per week) in determining serum 25-hydroxyvitamin D (25(OH)D) and ferritin concentrations among pregnant women in Hong Kong, China. We hypothesized that the association between dietary patterns and serum ferritin concentrations will remain significant after controlling vitamin D supplementation frequency, whereas regular vitamin D supplementation will compensate for inadequate dietary intake to improve serum 25(OH)D concentrations during pregnancy.

## 2. Method

### 2.1. Participants

All women making a visit to the antenatal clinic of local public hospitals during the period of July 2019 to December 2020 were invited to participate if they were healthy, Hong Kong Chinese citizens, aged 18 or above, at 25 to 35 weeks gestation, residing in Hong Kong, and literate in Chinese.

### 2.2. Design and Procedure

Data collection took place in the waiting area of the antenatal clinic. Upon providing written informed consent, participants completed demographic questionnaires including questions about the weekly frequency of vitamin D supplementation during pregnancy. The electronic version of the Food Frequency Questionnaire (eFFQ) was also administered. In addition, their peripheral blood samples were collected by trained phlebotomists. As a token of appreciation for their participation in this study, an incentive of HKD 50 supermarket voucher was offered to the participants at the end of the session. The research protocol was approved by the Institutional Review Board of the University of Hong Kong/Hospital Authority Hong Kong West Cluster Research Ethics Committee (UW 13-055).

### 2.3. Measures

#### 2.3.1. Dietary Patterns

Participants completed the eFFQ to report their food intake over the past month. Details of the eFFQ development can be found in the previous publication [10]. Briefly, the eFFQ is a modified version of the original FFQ, which was validated in the general adult population [11]. A total of 311 food items were categorized into twelve food groups, namely fish and seafood, mushrooms, eggs, dairy beverages, beans, fruits, grains, meats, snacks, soups, vegetables, and condiments and oil. Frequency options include once a month, 2–3 times per month, once to twice a week, 3–4 times per week, 5–6 times per week, and every day. Portion size was reported freely using either standardized household measurements or gram weight for food items. It was then converted into the number of daily servings according to the local guidelines [9]. In this study, the identification of latent subgroups was based on the categorization of whether the recommended number of daily servings of vegetables, fruits, meat, fish, eggs, or alternatives was met. Grains and dairy food groups were excluded because grains are a food staple in the Chinese diet, whereas dairy consumption levels are generally low for people living in Hong Kong [12]. Thus, these food groups were not considered helpful for differentiating dietary classes in this study.

#### 2.3.2. Serum 25(OH)D and Ferritin Concentrations

Serum was extracted from the collected peripheral blood samples. In this study, the liquid chromatography-tandem mass spectrometry (LC-tandem MS) method was adopted to determine serum 25(OH)D concentration, defined as the sum of 25(OH)D3 and 25(OH)D2 minus 3-Epi-25(OH)D3, using the QTRAP 5500 LC-MS/MS system (AB SCIEX Instruments, Framingham, MA, USA). The method has been verified against samples from the Vitamin D External Quality Assessment Scheme with satisfactory performance (within ± 15% of the target value) [13]. Blood serum samples were also tested for ferritin by a local accredited laboratory.

#### 2.3.3. Weekly Vitamin D Supplementation Frequency

Participants were asked, “how often do you take vitamin D supplements over the course of your current pregnancy?”. The answer options provided included “everyday”, “6–4 times per week”, “1–3 times per week”, “less than 1 time per week”, and “never”. Participants who selected “everyday”, “6–4 times per week”, and “1–3 times per week” were grouped as the “at least 1 time per week” subgroup, whereas those who selected “less than 1 time per week” and “never” were grouped as the “less than 1 time per week” subgroup.

#### 2.3.4. Demographics

Demographics, including chronological age, gestational age, gravidity, parity, marital and employment status at enrollment, highest education level, monthly family income, and history of chronic diseases, were self-reported by the participants.

### 2.4. Data Analysis

All data were analyzed using both the SPSS statistical software package (Version 26.0, SPSS Inc., Chicago, IL, USA) and the R statistical software (version 4.1.1, R Core Team, Vienna, Austria). Descriptive statistics were calculated to summarize the characteristics of participants and their families. A series of independent *t*-tests (for continuous variables) and chi-square analyses (for categorical variables) were performed to determine whether demographics and pregnancy characteristics, including vitamin D supplementation patterns and serum 25(OH)D and ferritin concentrations, differed between the dietary pattern groups. Data of ferritin were normalized using log transformation. There were no missing values on any of the key variables. Assumptions of linear regression including normality and linearity were assessed and verified graphically. There was no evidence of outliers and multicollinearity between variables. Latent class analysis (LCA) was first applied to identify latent subgroups of pregnant women with similar dietary patterns as indicated by the dichotomous status (sufficient/insufficient) of the three food group parameters using the R package “poLCA” (R Core Team, Vienna, Austria) [14]. Simple models with fewer classes were preferred and selected based on statistical fit indices (Akaike Information Criteria (AIC), Bayesian Information Criteria (BIC), adjusted BIC (aBIC), and consistent AIC (cAIC)) [15]. Lower values indicate better fitting models. The associations of vitamin D supplementation frequency and dietary patterns with serum 25(OH)D and ferritin concentrations were tested using hierarchical linear regression analyses [16]. The first model (Model 1) included the main categorical variable “inadequate dietary intake” and the covariate “age at assessment”. In the next model (Model 2), the other main categorical variable “infrequent vitamin D supplementation” was added. In Model 3, the interaction term “diet × supplementation” was created and included in the adjusted regression models. All regression coefficients were unstandardized, with *p*-values < 0.05 denoting statistical significance.

## 3. Results

### 3.1. Sample Characteristics

The overall sample consisted of 572 women (average age at assessment: 34 years) at 26 weeks gestation on average. Table 1 shows their demographic and pregnancy characteristics. The majority of women were pregnant for the first time as indicated by the average gravidity of 1.80 and parity of 0.62. In the study sample, 500 (90.9%) had no history of chronic diseases. 325 (57.3%) had completed tertiary education, and 367 (75.7%) were either full or part-time employees at the time of assessment. In addition, 276 (48.2%) had inadequate dietary intake, whereas 296 (51.8%) had adequate dietary intake. The adequate and inadequate dietary intake subgroups had similar demographic characteristics and serum 25(OH)D and ferritin concentrations, but the subgroups differed in terms of vitamin D supplementation frequency (*p* = 0.01), with a higher proportion of pregnant women in the inadequate dietary intake subgroup (27.5%) taking supplements less than once per week compared to those in the adequate dietary intake subgroup (18.6%). The number of participants with vitamin D deficiency (<50 nmol/L) was low in both subgroups (inadequate: 21 (7.6%); adequate: 14 (4.7%)).

### 3.2. Model Selection and Latent Subgroups

Models with one through four latent classes were compared in order to select a model of multiple food groups. Table 2 shows the values of the information criteria. The statistics suggested the two-class model (AIC = 2249.30 for two latent classes versus AIC = 2257.30 for three latent classes; BIC = 2279.74 for two latent classes versus BIC = 2305.14 for three latent classes; cAIC = 2286.74 for two latent classes versus cAIC = 2316.14 for three latent classes; and aBIC = 2257.52 for two latent classes versus aBIC = 2270.22 for three latent classes), and its parameter estimates also presented a solution with a logical, substantive interpretation. Each latent class corresponds to an underlying subgroup of pregnant women characterized by a particular pattern of food consumption. The parameter estimates shown in Table 3 provide the necessary information for interpreting and labeling each diet subgroup. Specifically, the first latent class labelled Inadequate is characterized by a high probability of having insufficient servings of vegetables (0.85), fruits (0.70), or meat, fish, eggs, or alternatives (0.62), whereas the second latent class was labelled Adequate due to the low probability of pregnant women in this subgroup reporting insufficient servings of the three food groups.

### 3.3. Associations of Vitamin D supplementation Frequency and Dietary Patterns with Serum 25(OH)D and Ferritin Concentrations among Pregnant Women

**Figure 1:**
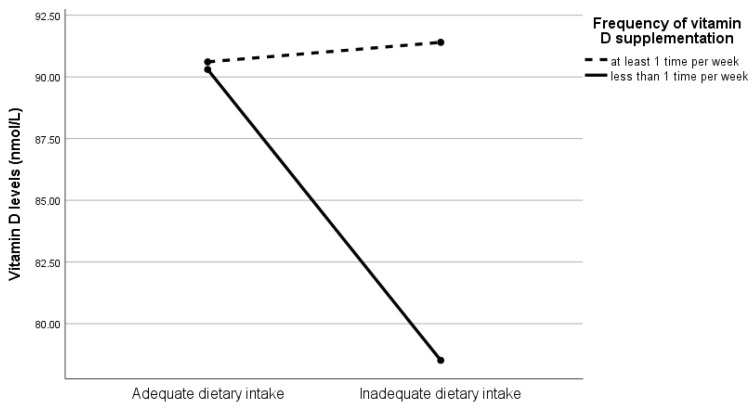
Serum 25(OH)D concentrations by status of dietary patterns and vitamin D supplementation frequency. 25(OH)D, 25-hydroxyvitamin D.

Serum 25(OH)D and ferritin concentrations had a small yet statistically significant correlation with each other (*r* = 0.14, *p* < 0.01). Table 4 shows the estimates of associations of dietary patterns and vitamin D supplementation frequency with serum 25(OH)D and ferritin concentrations after adjusting for age at assessment. There was a significant association between dietary patterns and serum ferritin concentrations among pregnant women, with lower serum ferritin concentrations found in the inadequate dietary intake subgroup compared to the adequate dietary intake subgroup (β = −0.06, *p* = 0.03). This association remained significant after further adjusting for vitamin supplementation frequency (β = −0.05, *p* = 0.04). No significant interaction between vitamin D supplementation frequency and dietary patterns on serum ferritin concentrations was found. On the other hand, although serum 25(OH)D concentrations had no association with dietary patterns, its association with vitamin D supplementation frequency remained significant after adjusting for dietary patterns, with lower serum 25(OH)D concentrations observed in those who took vitamin D supplements less than once a week compared to the more frequent supplementation group (β = −6.89, *p* = 0.01). The results of moderation analysis showed that only the interaction between vitamin D supplementation frequency and dietary patterns was significant (β = −13.22, *p* = 0.01). As illustrated in Figure 1, the differences in serum 25(OH)D concentrations between the groups of vitamin D supplementation frequency were trivial when the dietary intake amount was adequate, but for those with inadequate dietary intake, serum 25(OH)D concentrations were higher among pregnant women who took vitamin D supplements at least once a week compared to those who did not.

## 4. Discussion

In this study, latent class analysis techniques were employed to identify subgroups of pregnant women in Hong Kong based on their dietary patterns during pregnancy. Results revealed two dietary patterns, which were labelled as adequate and inadequate dietary intake subgroups, respectively. The adequate dietary subgroup had a high probability of meeting the recommended number of daily servings for all three target food groups (vegetables; fruits; and meat, fish, eggs, or alternatives), whereas the inadequate dietary subgroup had a high probability of not meeting the recommendations. A previous study of 285 healthy pregnant women in Ireland also identified two dietary patterns [17]. The unhealthy diet cluster consisting of 124 women (43.5%) reported significantly higher median intakes of white bread, refined breakfast cereals, confectionery, chips, processed meats, and high-energy beverages; the health-conscious diet cluster consisting of 161 women (56.5%) reported significantly higher intakes of wholegrain breads and breakfast cereals, fruits, vegetables, fruit juice, fish, low-fat milk, and white meats. Another study found that no pregnant women in Australia had achieved the daily food group recommendations provided in the Australian Guide to Healthy Eating [18]. Overall findings suggest that pregnant women, regardless of geographical locations, tend to make consistent food choices (either sufficient or insufficient intake). Considering the adverse health consequences of inadequate dietary intake for mothers and their offspring [19,20], antenatal nutrition counseling together with dietary monitoring and education services should be provided to pregnant women, particularly for those who show signs of inadequate dietary intake.

Given that previous research has focused on either food intake or supplementation but not both, this study expands the scope of previous research by demonstrating a link between food preferences and use of vitamin supplements during pregnancy. We found that although the adequate and inadequate dietary intake subgroups had similar demographic backgrounds, the adequate dietary intake subgroup was more likely to have regular vitamin D supplementation (i.e., at least 1 time per week) than the inadequate dietary intake subgroup. This finding can be explained partly by factors such as motivation to take supplements and knowledge about healthy diets, all of which can influence supplementation frequency. Dietary supplements are intended to supplement the diets of people who eat insufficiently. However, people who eat insufficiently tend to have low health consciousness. It has been posited that pregnancy is a time when women have increased motivation to make dietary improvements [21]. Research has found that pregnant women receiving dietary advice, regardless of the frequency of prenatal visits and sociodemographic characteristics, had a higher probability of using multivitamins compared to those lacking information [22]. The attendance of prenatal education sessions was also related to supplement intake [23]. Hence, healthcare professionals play an important role in explaining to pregnant women whether taking dietary supplements is necessary for improving their nutrient intake.

In addition, we found that the inadequate dietary intake subgroup had lower serum ferritin concentrations compared to the adequate dietary intake subgroup, after adjusting for their vitamin D supplementation frequency status. The findings are consistent with previous studies reporting that vitamin D supplementation cannot improve serum ferritin concentrations in pregnant women [5,6,7]. In addition, since serum ferritin is a recognized indicator of iron status, our findings highlight the implications of dietary patterns in determining the risk of iron deficiency. In this study, the adequate dietary intake subgroup was more likely than the inadequate dietary intake subgroup to meet the recommended daily servings of vegetables, fruits, meats, fishes, eggs, and other alternatives. Red meat is the major source of dietary intake of heme iron, which represents more than 95% of functional iron in the human body [24]. A previous review documented evidence showing the positive association between meat consumption and iron status among young women [25]. Vitamin C (ascorbic acid) in fresh fruits and vegetables can also act as an enhancer to promote iron solubility and absorption through the conversion of iron chelators and ferric iron to ferrous iron [26,27]. On the other hand, our findings suggest that the association of dietary patterns with serum 25(OH)D concentrations may be conditional upon the frequency of vitamin D supplementation. As reported by previous studies [28,29,30,31,32,33], individuals with low baseline serum vitamin D levels could have greater responses to vitamin D supplementation than those with higher baseline serum vitamin D levels. Therefore, regular vitamin D supplement use is particularly important for pregnant women who eat insufficiently to achieve sufficient vitamin D levels.

This study has several limitations. First, while the participants were recruited from different Hong Kong local hospitals, they were not fully representative of Hong Kong’s pregnant women. However, the cluster analysis identified two latent subgroups which are largely consistent with previous research on this topic. Second, the number of participants who were vitamin D deficient in our sample was small, and other lifestyle factors (e.g., physical activity and smoking) were not assessed in this study. The findings thus may not be applicable to special subgroups such as those who lack vitamin D due to genetic factors or those who smoke. Third, dietary patterns and vitamin D supplementation frequency were self-reported by participants using questionnaires. Their reports may have recall bias. Fourth, only vitamin D supplementation frequency was examined. Future research should assess the dosage and frequency of other supplements such as multivitamins and iron supplements. In addition, more studies should be conducted to identify the best dietary composition for achieving the daily recommended dietary intake of iron and vitamin D.

## 5. Conclusions

This study expands the current understanding of vitamin D supplementation use by showing the relative importance of dietary patterns and vitamin D supplementation frequency for pregnant women. It also replicates previous research demonstrating that dietary sources are more important than vitamin D supplementation for promoting ferritin expression. The findings are particularly useful for allocation of limited health care resources. They point to the need of simple dietary screening in early pregnancy and targeted nutrition counseling and education services at antenatal clinics to improve the dietary knowledge and behavior of at-risk pregnant women such as those who have inadequate dietary intake. More studies are needed to examine the misconceptions of food safety and nutrition in these women and their behavioral barriers to meeting dietary recommendations in pregnancy.

## Figures and Tables

**Table 1:** Participant characteristics.

	Overall (*n* = 572)	Inadequate Dietary Intake (*n* = 276)	Adequate Dietary Intake (*n* = 296)	*p*-Value
Chronological age (years), mean (SD)	34.09 (3.98)	34.05 (3.96)	34.12 (4.01)	0.84
Gestational age (weeks), mean (SD)	26.03 (4.81)	26.26 (4.18)	25.82 (5.33)	0.27
Gravidity, mean (SD)	1.80 (1.04)	1.79 (1.02)	1.81 (1.07)	0.83
Parity, mean (SD)	0.62 (0.79)	0.60 (0.77)	0.65 (0.81)	0.44
Marital status, *n* (%) ^a^				
Married	536 (94.4)	258 (94.2)	278 (94.6)	0.84
Single/divorced/widowed	32 (5.6)	16 (5.8)	16 (5.4)	
Highest education level, *n* (%) ^a^				0.24
Lower secondary education or below	27 (4.8)	17 (6.2)	10 (3.4)	
Upper secondary education/higher certificate/diploma	215 (37.9)	106 (38.7)	109 (37.2)	
Bachelor’s degree or above	325 (57.3)	151 (55.1)	174 (59.4)	
Occupation, *n* (%) ^a^				0.54
Housewife	93 (19.2)	42 (17.3)	51 (21.1)	
Full-/part-time employment	367 (75.7)	189 (77.8)	178 (73.6)	
Unemployment	25 (5.2)	12 (4.9)	13 (5.4)	
Monthly family income (HKD ’000), mean (SD)	51.3 (33.6)	49.6 (30.9)	52.8 (36.0)	0.27
History of chronic diseases, *n* (%) ^a^				0.98
Yes	50 (9.1)	24 (9.1)	26 (9.1)	
No	500 (90.9)	241 (90.9)	259 (90.9)	
Weekly vitamin D supplementation frequency, *n* (%) ^a^				0.01
At least 1 time per week	427 (77.2)	190 (72.5)	237 (81.4)	
Less than 1 time per week	126 (22.8)	72 (27.5)	54 (18.6)	
Vitamin D level (nmol/L), mean (SD)	89.00 (26.30)	87.83 (27.08)	90.03 (25.56)	0.32
Log10 ferritin (pmol/L), mean (SD)	1.79 (0.30)	1.76 (0.29)	1.81 (0.30)	0.08

^a^ Missing data not shown. SD, standard deviation,

**Table 2:** Indicators of it for models with one through three latent classes.

Number of Classes	BIC	AIC	aBIC	cAIC
1	2365.926	2352.878	2356.402	2368.926
2	2279.744	2249.301	2257.523	2286.744
3	2305.141	2257.301	2270.221	2316.141

BIC, Bayesian Information Criteria; AIC, Akaike Information Criteria; aBIC, adjusted BIC; cAIC, consistent AIC.

**Table 3:** Parameter estimates for each latent class.

Food Group	Class 1	Class 2
	Inadequate Dietary Intake	Adequate Dietary Intake
	(*n* = 276, 48.2%)	(*n* = 296, 51.8%)
Vegetables (insufficient servings/day)	0.85	0.25
Fruits (insufficient servings/day)	0.70	0.19
Meat, fish, eggs, or alternatives (insufficient servings/day)	0.62	0.17

**Table 4:** Associations of vitamin D supplementation frequency and diet status with serum 25(OH)D and ferritin concentrations among pregnant women.

	Ferritin		25(OH)D	
	*β* (95% CI)	*p*-Value	*β* (95% CI)	*p*-Value
Model 1				
Inadequate dietary intake	−0.06 (−0.01, −0.11)	0.03	−2.57 (−6.99, 1.84)	0.25
Model 2				
Inadequate dietary intake	−0.05 (−0.004, −0.10)	0.04	−1.97 (−6.38, 2.45)	0.38
Infrequent vitamin D supplementation	−0.03 (0.03, −0.09)	0.39	−6.89 (−12.15, −1.63)	0.01
Model 3				
Inadequate dietary intake	−0.04 (−0.09, 0.02)	0.22	1.03 (−3.97, 6.03)	0.69
Infrequent vitamin D supplementation	0.02 (−0.07, 0.11)	0.70	0.32 (−7.45, 8.08)	0.94
Vitamin D × Diet	−0.08 (−0.20, 0.04)	0.19	−13.22 (−23.73, −2.71)	0.01

All models were adjusted for age at assessment. 25(OH)D, 25-hydroxyvitamin D; CI, confidence interval.

## Data Availability

The data that support the findings of this study are available upon request from the corresponding author.
